# Supplementary material for: The neural computation of inconsistent choice behavior
Source: Nat Commun. 2019 Apr 5;10:1583. doi: 10.1038/s41467-019-09343-2 (PMC6450930; doi:10.1038/s41467-019-09343-2)
Supplement: Supplementary file 1 — Supplementary Information [file 41467_2019_9343_MOESM1_ESM.pdf]

The Neural Computation of Inconsistent Choice Behavior

Kurtz-David et al.

Supplementary Information

Supplementary Notes

Supplementary Note 1: The General Axiom of Revealed preference (GARP) and aggregate inconsistency indices.

A useful equivalent description of the experiment: The Decision Maker has an initial endowment E and she is faced with two types of products – one (x_1) is a security that realizes if a fair coin falls on Heads and the other (x_2) is a security that realizes if the same fair coin falls on Tails. Each type of security is assigned with a price (p_1 and p_2). In each trial the Decision Maker is required to spend all her endowment on the purchase of the two types of securities. The trials differ by the securities' prices and the Decision Maker's endowment. The prices and endowment are drawn randomly.

Budget sets and budget lines: In each decision problem the subject can choose every affordable bundle (pair of quantities of products x_1 and x_2). We call the set of all affordable bundles: the budget set. In this experiment the subjects are forced to choose a bundle that exhausts all the Decision Maker's endowment. We call the set of all affordable bundles that exhaust the endowment: the budget line, and formally, $p_1x_2 + p_2x_2 = E$ (or $\mathbf{px} = E$). Hence, the Decision Maker's problem in our experiment can be described as a choice of a bundle of securities from a linear budget line. Supplementary Fig. 2a exhibits three budget lines that represents three typical trials the subjects faced during the experiment.

The experimental data set: For each individual the data set contains typically 108 observations. Each observation i includes the chosen bundle $\mathbf{x}^i$ (that includes x_1^i and x_2^i) and the prices assigned to each security in that trial $\mathbf{p}^i$ (p_1^i and p_2^i). We denote the data set by **D**.

Direct Revealed Preference Relations[1,2]: We say that an observed bundle x^i is directly revealed preferred to another bundle $\mathbf{x}$, if $\mathbf{x}^i$ was chosen while $\mathbf{x}$ was available ($\mathbf{p}^i\mathbf{x}^i \geq \mathbf{p}^i\mathbf{x}$) and we denote it by $\mathbf{x}^i R_D^0\, \mathbf{x}$. For example, in Supplementary Fig. 2a bundle $\mathbf{x}^2$ was chosen, while bundle $\mathbf{x}^1$ was available (both bundles reside on the same budget line), and hence $\mathbf{x}^2$ is revealed preferred to $\mathbf{x}^1$, $\mathbf{x}^2 R_D^0\, \mathbf{x}^1$.
We say that an observed bundle $\mathbf{x}^i$ is strictly directly revealed preferred to another bundle $\mathbf{x}$ if $\mathbf{x}^i$ was chosen while $\mathbf{x}$ was strictly available ($\mathbf{p}^i\mathbf{x}^i > \mathbf{p}^i\mathbf{x}$) and we denote it by $\mathbf{x}^i P_D^0\, \mathbf{x}$. For example, in Supplementary Fig. 2a, bundle $\mathbf{x}^3$ is strictly revealed preferred to bundle $\mathbf{x}^2$, as $\mathbf{p}^3\mathbf{x}^3 > \mathbf{p}^3\mathbf{x}^2$.

Revealed Preference Relations: Consider a sequence of bundles such that each bundle is directly revealed preferred to the next. We say that an observed bundle $\mathbf{x}^i$ is revealed preferred to another bundle $\mathbf{x}$ (denoted $\mathbf{x}^i R_D \mathbf{x}$) if there exists a sequence of observed bundles ($\mathbf{x}^j, \mathbf{x}^k, \dots, \mathbf{x}^m$), that are directly revealed preferred to one another and $\mathbf{x}^i R_D^0\, \mathbf{x}^j$ and $\mathbf{x}^m R_D^0\, \mathbf{x}$. In Supplementary Fig. 2a, $\mathbf{x}^3 R_D \mathbf{x}^1$ as we observe the sequence $\mathbf{x}^3 R_D^0\, \mathbf{x}^2$ and $\mathbf{x}^2 R_D^0\, \mathbf{x}^1$.

Consistency and the General Axiom of Revealed Preference (GARP): A Dataset **D** satisfies GARP if and only if $\mathbf{x} R_D \mathbf{y}$ implies not $\mathbf{y} P_D^0\, \mathbf{x}$ (If the data reveals that **x** is preferred over **y**, **y** cannot, at the same time, be chosen when **x** is strictly available). A decision maker is said to be consistent if her choices do not exhibit any strict revealed preference cycles, meaning, if she satisfies GARP. Afriat Theorem[2] shows that a decision maker is consistent if and only if her choices can be represented as a maximization of a (well-behaved) utility function. In Supplementary Fig. 2b, we can

elicit a utility function that is compatible with the subject's choices, as the subject's choices satisfy GARP.

The Extent of Inconsistency: The experimental and empirical literature in Economics and Psychology frequently reports that many subjects fail GARP, meaning they are classified as inconsistent and therefore, by Afriat Theorem, there is no (non-satiated) utility function that represents their preferences. As a result, a literature that measures the extent of inconsistency evolved by relaxing the revealed preference relations defined above.

Relaxed Revealed Preference Relations: The standard direct revealed preference relation states that the chosen bundle is preferred over all available bundles. One way to relax this assertion is to define for every observation i a constant $v_i \in [0,1]$ and say that an observed bundle x^i is v^i-directly revealed preferred to another bundle $\mathbf{x}$ if $\mathbf{x}^i$ was chosen while $\mathbf{x}$ was available even if the endowment was only a fraction v^i of its real value ($\mathbf{p}^i\mathbf{x}^i$). We refer to v^i as the adjustment of the budget line of observation i and denote the vector of all adjustments by $v \in [0\ 1]^n$. Thus, given $v \in [0\ 1]^n$, an observed bundle $\mathbf{x}^i$ is v-directly revealed preferred to another bundle x if $v^i\mathbf{p}^i\mathbf{x}^i \geq \mathbf{p}^i\mathbf{x}$ and we denote it by $\mathbf{x}^i R^0_{D,v}\ \mathbf{x}$. Similarly, given $v \in [0\ 1]^n$, an observed bundle $\mathbf{x}^i$ is v-strictly directly revealed preferred to another bundle $\mathbf{x}$ if $v^i\mathbf{p}^i\mathbf{x}^i > \mathbf{p}^i\mathbf{x}$ and we denote it by $\mathbf{x}^i P^0_{D,v}\ \mathbf{x}$. Also, given $v \in [0\ 1]^n$, an observed bundle $\mathbf{x}^i$ is v-revealed preferred to another bundle $\mathbf{x}$ (denoted $\mathbf{x}^i R_{D,v}\mathbf{x}$) if there exists a sequence of observed bundles ($\mathbf{x}^j, \mathbf{x}^k, \dots, \mathbf{x}^m$), that are v-directly revealed preferred to one another and $\mathbf{x}^i R^0_{D,v}\ \mathbf{x}^j$ and $\mathbf{x}^m R^0_{D,v}\ \mathbf{x}$.

The Relaxed General Axiom of Revealed Preference (GARP$_v$): We relax the definition of GARP to test whether a dataset is consistent given some adjustments to the budget lines. Formally, given $v \in [0\ 1]^n$, Dataset **D** satisfies GARP$_v$ if and only if $\mathbf{x}R_{D,v}\mathbf{y}$ implies not $\mathbf{y}P^0_{D,v}\mathbf{x}$.

Aggregate inconsistency indices. As subjects often violate GARP, and are inconsistent[3–7], one would like to measure their level of inconsistency. The simplest way would be to count the number of GARP violations. Other well-known non-parametric inconsistency indices are Afriat index, Varian index and Houtman-Maks index (see below). For each subject, we calculated the number of GARP violations and Afriat index for the entire experiment (108 trials). We were unable to compute Varian Index and Houtman-Maks Index at the aggregate level as they are hard computationally[8,9] (see Appendix B in Halevy et al. (2018)[7]).

Varian Index[8]: Varian proposed to measure the extent of inconsistency by the minimal adjustment needed to satisfy GARP. In order to aggregate the elements of $v \in [0\ 1]^n$ (the vector of adjustments per observation) an aggregator function denoted $f(v)$ is required (e.g. minimum, mean, sum of squares, etc.). This function is such that if v is the vector of 1s (no adjustments), its value is zero and it increases when v decreases. Formally, the Varian index of Dataset **D**, given aggregator *f*, is

$$(1)\quad I_V(\mathbf{D}, f) = \inf_{v \in [0\ 1]^n : \mathbf{D} \text{ satisfies GARP}v} f(v)$$

In Supplementary Fig. 2d-e we show Varian Index with two different adjustments vectors v. The adjusted budget sets represented by the dashed lines in each figure. The minimal adjustment vector is chosen according to the aggregator functions $f(v)$ in use.

Afriat Index (The Critical Cost Efficiency Index, CCEI)[1,10]: A specific case of Varian index, where the aggregator function is the minimum function. Halevy et al. (2018)[7] show that it is equivalent to calculating the minimal *uniform* adjustment needed to satisfy GARP. Supplementary

Fig. 2f depicts Afriat Index, the elements in v are identical (we adjust each budget set by the same percentage) and represented by the dotted lines.

Houtman-Maks Index[11]**:** The maximal subset of D that satisfies GARP (normalized by the size of **D**). Halevy et al. (2018)[7] show that it is equivalent to restricting Varian Index calculation to take into account only binary adjustments (adjust completely or not adjust at all) and use the summation aggregator.

Supplementary Note 2: Evidence that the subjects in our sample understood the experimental task

We had considerable evidence that the subjects understood the task and did not behave randomly. Subjects exhibited substantially fewer GARP violations and much lower inconsistency indices then simulated random decision makers (Fig. 3a).[12]

Moreover, a subject who violates first order stochastic dominance (FOSD) is expected to allocate more tokens to the expensive account, rather than the cheaper account. In our dataset, we find a total of 337 trials (9.5% of trials) with FOSD violations. However, if we allow the subject to allocate the cheaper product up to 90% of her allocation to the expensive product, then the number of FOSD violations drops significantly to only 66 trials (1.8% of trials). Such a low amount of FOSD violations indicates subjects understood the task indeed.

In addition, all subjects obeyed the law of demand (even those who were highly inconsistent), meaning they reduced the number of tokens they allocated to the Y-account as its relative price increased (represented by the budget line slope). The predominant patterns in behavior are depicted in Fig. 3b. See Supplementary Fig. 7 for scatterplots of all subjects.

Finally, behavior within the fMRI is not substantially different from behavior in the standard behavioral laboratory setting. A comparison with the Choi et al. (2007) study reveals that the distributions of the Afriat inconsistency index,[1,10] are quite similar (Fig. 3d).

Supplementary Note 3: Controlling for changes in heuristics

We ensured that our results are not solely due to inconsistency in choice behavior over blocks of the experiment, but that inconsistency within each block was related to the mpFC and ACC activity. We therefore calculated our inconsistency index based on the parameters estimated from each block separately (27 trials in each block), henceforth termed Trial-specific-MMI-blocks. A verification check found a positive correlation between Trial-specific-MMI-blocks ($\beta=0.8468$, $p<0.0001$) and Trial-specific-MMI. Supplementary Figure 4a presents the estimated parameters for each subject in each block. Supplementary Table 1 presents a classification of choice behavior for each block for each subject. Only eight out of the thirty-three subjects in our sample appeared to change their choice behavior across blocks, with only 6 of these 8 subjects revealing a large average difference between Aggregate-MMI-blocks and Aggregate-MMI for the entire experiment. This suggests that only these subjects exhibited consistency within blocks but inconsistency over the entire experiment (Supplementary Table 2, Supplementary Figure 4b).

The GLM analysis was repeated twice using: (a) Trial-specific-MMI-blocks as the trial-specific inconsistency regressor for all subjects, (b) a combined regressor of Trial-specific-MMI-blocks for the eight subjects whose parameters changed dramatically across blocks and Trial-specific-MMI

for the rest of our sample. Both analyses revealed that choice inconsistency is correlated with mPFC and ACC activations (Supplementary Figs. 4c-d). Hence, our findings are robust even if we take into account subjects that are only inconsistent across blocks.

Supplementary Note 4: Controlling for misspecification of utility

Since the value of the MMI is constructed from both a misspecification and inconsistency elements (see Methods), we must ensure that the BOLD signals in the mPFC and dACC track the inconsistency index and are not correlated solely with the misspecification element. According to Halevy et al. (2018)[7], when changing the functional form of the utility function the inconsistency element remains unchanged, while the misspecification varies. Therefore, we repeated our analysis using a different utility function (see Methods). Importantly, in the neuroimaging analysis, the mPFC and ACC activations were still correlated with Trial-specific-MMI even when using the alternative utility formulation (Supplementary Figure 6b, $p<0.0005$, cluster-size corrected). This implies that activations in the mPFC and ACC are correlated with the severity of choice inconsistency on a trial-by-trial level, independent of the functional form of the utility function.

Supplementary Note 5: Orthogonality Analysis

We conducted an orthogonality analysis in the main GLM whereby we replaced Trial-specific-MMI with the residuals ($\tilde{e}$) from a regression of SV on Trial-specific-MMI (and similarly for SV). After orthogonalizing the two regressors, the overlap of the significant voxels in mPFC holds (Methods and Supplementary Figure 11a). In addition, we contrasted SV≠Trial-specific-MMI, using the same RFX-GLM reported in the whole-brain analysis. We found a dis-conjunct activity of the two regressors in the mPFC/ACC at the same locations that we report in the general GLM (Supplementary Figure 11b). This suggests that the activity in these brain areas representing SV and Trial-specific-MMI is indeed dissociated.

Supplementary Note 6: Experiment's Instructions (Translation from Hebrew)

Introduction
This is an experiment in decision making. Research grants have provided the funding needed for this experiment. Your payoffs will depend partly on your decisions, and partly on chance. Please pay careful attention to the instructions as a considerable amount of money is at stake.

Before the MRI scan, you will practice the experiment's software in front of a computer. The practice is expected to last 20 minutes. Soon after you will be requested to remove any metal object of your body, and then the MRI scan will begin. At the beginning of the scan we will conduct a short calibration of the MRI scanner, which will last a few minutes, and then the experiment will begin, and last about 60 minutes. At the end of the experiment, we will perform an anatomical scan, during which you will not be asked to perform any task. The anatomical task will take another 5 minutes. The entire scan should not exceed 75 minutes.

At the end of the experiment you will receive a payoff for your earnings in the experiment, as well as a show-up fee of 100 NIS for the time you dedicated to the experiment. Details of how you will make decisions and receive payments are described below.

During the experiment we will use the term of "tokens" instead of NIS. Your payoffs will be calculated in tokens and converted to NIS at the end of the experiment, according to a conversion rate of 1 token = 5 NIS.

The decision problem

In this experiment, you will be asked to answer 108 independent decision problems that share a common form, one decision problem after the other. This section describes in detail the process that will be repeated in all decision problems, and the computer program that you will use to make your decisions.

In each decision problem you will be asked to allocate tokens between two products, labeled *X* and *Y*. The *X*-Product is represented by the *X*-axis in a 2 dimensional graph, and the *Y*-Product is represented by the *Y*-axis, respectively. In each problem you will have to choose a point on the graph that represents a possible allocation of tokens. Examples of the lines you might face are depicted in Supplementary Fig. 13a.

At the end of the experiment one of the products will be chosen randomly. Each product has an equal chance of being casted.

In each decision problem you may choose any pairing of *X* and *Y* on the line. For example, as you can see in Supplementary Fig. 13b, choosing allocation A represents an allocation of 10 tokens to the *X*-product and 35 tokens to the *Y*-product. Meaning, by choosing allocation *A* you have a 50% chance of winning 10 tokens and a 50% chance of winning 35 tokens. If the *X*-product is casted, you win 50 NIS (10 tokens * 5 NIS per token = 50 NIS), while if the *Y*-product is casted you win 175 NIS (35 tokens * 5 NIS per token = 175 NIS).

Alternatively, another possible allocation is *B*, where 45 tokens are allocated to the *X*-product, and 10 tokens to the *Y*-product. That is, choosing allocation B means a 50% chance of winning 45 tokens, and a 50% chance of winning 10 tokens. If the *X*-product is casted, you win 225 NIS (45 tokens * 5 NIS per token = 225 NIS), while if product *Y* is casted you win 50 NIS (10 tokens * 5 NIS per token = 50 NIS).

Similarly, if you choose to allocate all tokens to the *X*-product (allocation *C*), then you have a 50% chance of winning nothing, and a 50% chance of winning 60 tokens equal to 300 NIS (60 tokens * 5 NIS per token = 300 NIS).

On the other hand, if you choose an allocation where *X* and *Y* are of equal amount (allocation *D*), then you win that amount for sure, regardless of the results of the lottery. In that case, you win 25.7 tokens equal to 128.5 NIS (25.7 tokens * 5 NIS per token = 128.5 NIS).

These are obviously just examples. You may choose any possible pairing of tokens on the graph. Be aware that you are able to only choose pairs of tokens on the black line.

At the beginning of each decision problem the computer will randomly cast a line, similar to the lines depicted in Figure 1, from the set of lines that intersect with at least one of the axes at 50 or more tokens, but with no intercept exceeding 100 tokens. The lines selected in the various trials are independent of each other.

After making a choice, you will be asked to make an allocation in another independent decision problem. This process will be repeated up until you complete all decision problems.

Technical instructions:
To choose an allocation, use the trackball to move the pointer on the screen and position it on the allocation you desire. At any point, you can see on the top of the screen the allocation that the pointer is at. When you are ready to make a decision, left-click the trackball to submit your chosen allocation. Note that you can choose only *X* and *Y* combinations that are on the line.

After the click, your decision will be recorded, and you won't be able to make any changes. You have 12 seconds to make a decision. If by the end of the 12-seconds you will not make any decision, the computer will notify you with the following message: "Pay attention! In this round you did not choose any allocation." Soon after you submit your answer, and/or at the end of the 12 seconds, you will move to a black screen. We shall ask you not to move the trackball during the black screen. Next, you will be asked to make an allocation in another independent decision. At the beginning of each decision problem, the pointer will appear at the center of the screen.

This process will be repeated until you complete all 108 decision problems. The experiment is divided to 4 blocks of 9.5 minutes each. The blocks' durations is predetermined, and is unchangeable. At the end of the fourth block round you will be informed that this part of the experiment has ended.

Other tasks
After you complete the main task, we will present you with two additional tasks. These tasks will not be part of the prize lottery.

At the first task, you will be presented with different black lines, on each of them a black dot will be shown at random. You will have to move the pointer towards the black dot, and left-click the trackball exactly when you reach it. You will have 6 seconds to reach the black dot. Please try to be as precise as possible. You will be presented with 27 such lines, and the task will last 5.5 minutes.

At the second task, you will be presented with other black lines, and the headline at the top of the screen will inform you what is the (*X*,*Y*) target coordinate you have to reach, as fast and as precise as possible. For example, if the notification at the top of the screen reads (20,50), then you need to scroll the pointer towards the coordinate (20,50). Please notice that the left number corresponds to the *X*-axis, and the right number corresponds to the *Y*-axis. Similar to the main task, at any point, you can see on the top of the screen the coordinates that the pointer is at. You will have 12 seconds to reach the target coordinate. You will be presented with 27 such lines, and the task will last 9.5 minutes.

Payoff
At the end of the experiment, you will randomly select one of the decision problems you answered in the experiment. Each problem has an equal chance of being casted. Notice that the practice you will have outside the MRI scanner is not included in the lottery, and you will not receive a payoff for it.

Your payoff will be determined by the tokens-amount you allocated to the *X*-product, and by the tokens-amount you allocated to the *Y*-product. In another lottery, you will cast the winning product, *X* or *Y*, where each product has an equal chance of being casted.

You will win only the tokens you allocated to the product you casted. This amount will be converted to NIS. Please remember that each token equals 5 NIS. In case you cast a decision problem where no choice was made, the computer will randomly select an allocation for you.

If in the casted decision problem you chose the bundle (37,80), and the *X*-product was chosen, then you earn 37 tokens. The monetary value of 37 points is 185 NIS (37 points * 5 NIS per token = 185 NIS). On the other hand, had the *Y*-product been casted, you would have earned 80 tokens, which are equal to 400 NIS (80 tokens * 5 NIS per token = 400 NIS).

Your payoff is therefore composed of two elements: the monetary value of your winning prize in the experiment and a 100 NIS participation fee. You will receive the payment when you complete the experiment and exit the MRI scanner.

General
Your participation in the experiment and any information about your identity is subject to the information that appeared in the consent forms, and according to the decisions and procedures of Helsinki Committee. Moreover, any information about the payoffs you received will be kept strictly confidential.

Thank you and good luck!

Supplementary Note 7: Pre-scan questionnaire

There are 4 questions in the questionnaire, aiming to verify your complete understanding of the experiment's instructions. When you finish the questionnaire, please present your answers to the experimenter.

Question 1: please identify the intersections with the axes in the graphs depicted in Supplementary Fig. 14a:

1. Intersection with the *X*-axis: ___________
   Intersection with the *Y*-axis: ___________

2. Intersection with the *X*-axis: ___________
   Intersection with the *Y*-axis: ___________

Question 2: For each graph in Supplementary Fig. 14b, please identify the product that provides you with more tokens.

Question 3: The graphs in Supplementary Fig. 14c are examples for the deicsion problems in the experiment. These are just examples to verify you understood the experiment's instructions. In each example, please choose a possible allocation of *X* and *Y*. Please explain what are the possible payoffs for this allocation.

Question 4: Supplementary Table 8 summarizes the *X* and *Y* allocations of one of the subjects in the experiment. Besides the table are shown possible winning notifactions. Please answer the foolowing question:
a. What is tokens-amount the subject won?
b. What is the monetary value of the winning prize?

First possible winning notifaction:
"In this game the winning round is 2, and the fair coin tossed the *Y*-prodcut."

a. What is the token-amount the subject won? _______
b. What is the monetary value (in NIS) of the winning prize? _______

Second possible winning notifaction:
"In this game the winning round is 11, and the fair coin tossed the *Y*-prodcut."

a. What is the token-amount the subject won? _______
b. What is the monetary value (in NIS) of the winning prize? ______

Third possible winning notifaction:
"In this game the winning round is 15, and the fair coin tossed the *X*-prodcut."

a. What is the token-amount the subject won? _______
b. What is the monetary value (in NIS) of the winning prize? _______

Supplementary Figures

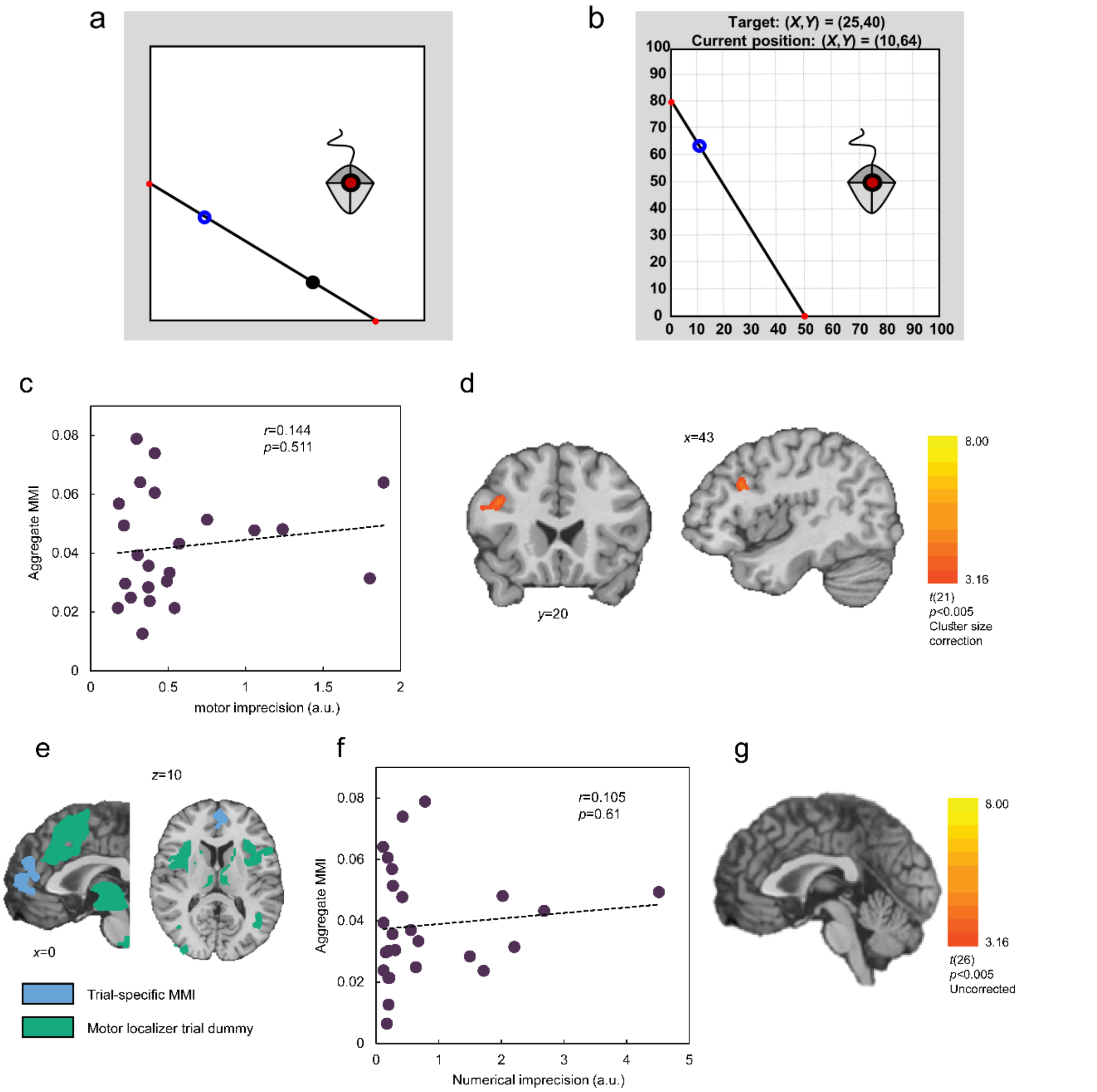

Supplementary Figure 1 | Functional localizers. Subjects completed two functional localizers at the end of the main task to eliminate alternative explanations for the sources of choice inconsistency. (a) A visualization of the motor localizer. Subjects had to move the blue cursor to the black target, using a trackball. (b) A visualization of the numerical localizer. Subjects had to reach the target coordinates. (c-d) Choice inconsistency is not generated by motor imprecision (“trembling hand”). (c) Average motor imprecision per subject was not correlated with Aggregate MMI scores (*r*=0.144, *p*=0.511). (d) The neural correlates of motor imprecision. RFX GLM, *n*=22, *p*<0.005, cluster size correction. *x*=43 and *y*=20 (MNI coordinates). Model regression: $\text{BOLD} = \beta_0 + \beta_1\text{RT} + \beta_2\text{Motor imprecision} + \beta_3\text{Slopes} + \varepsilon$. (e) Motor movements compared to Trial-specific-MMI. Motor movement is captured by the trial dummy of the motor functional localizer. RFX GLM, *n*=22, *p*<0.0005, cluster size correction. x=0 (MNI coordinates). Model regression: $\text{BOLD} = \beta_0 + \beta_1\text{RT} + \beta_2\text{Motor imprecision} + \beta_3\text{Slopes} + \varepsilon$. (f-g) Choice inconsistency is not generated by numerical imprecision. (f) The average numerical imprecision per subject was not correlated with the Aggregate MMI (*r*=0.105, *p*=0.61). (g) We did not find any neural correlates for the numerical imprecision at a corrected threshold. RFX GLM, *n*=27, *p*<0.005, not corrected. *x*=0 (MNI coordinates). Model regression: $\text{BOLD} = \beta_0 + \beta_1\text{RT} + \beta_2\text{Numerical imprecision} + \beta_3\text{Slopes} + \varepsilon$.

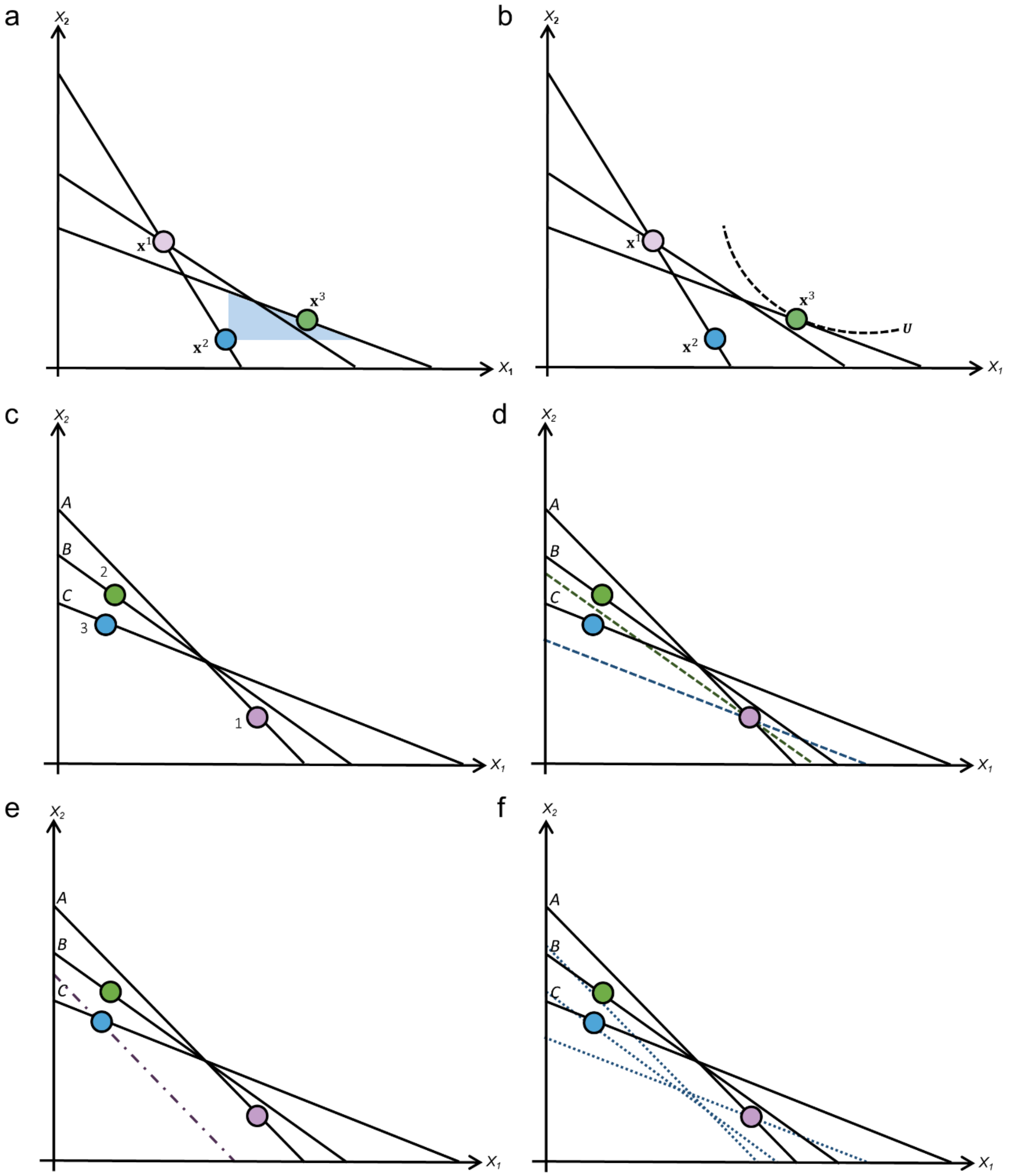

Supplementary Figure 2 | GARP and inconsistency indices. (a) Revealed preference relations with two accounts (x_1, x_2) and 3 choices $(\mathbf{x}^1, \mathbf{x}^2, \mathbf{x}^3)$. (b) Afriat Theorem and The General Axiom of Revealed Preference (GARP) for the same choices. (c) GARP violations with 2 products (x_1, x_2) and 3 choices. A total of 3 violations: Bundles $\mathbf{x}^1$ and $\mathbf{x}^2$ and Bundles $\mathbf{x}^1$ and $\mathbf{x}^3$ violate GARP using only direct relations, while Bundles $\mathbf{x}^2$ and $\mathbf{x}^3$ violate GARP since $\mathbf{x}^2$ is preferred to $\mathbf{x}^3$ (through $\mathbf{x}^1$) but $\mathbf{x}^3$ is strictly directly revealed preferred to $\mathbf{x}^2$. (d-e) Varian Index for the example depicted in (c). (f) Afriat Index for the example depicted in (c).

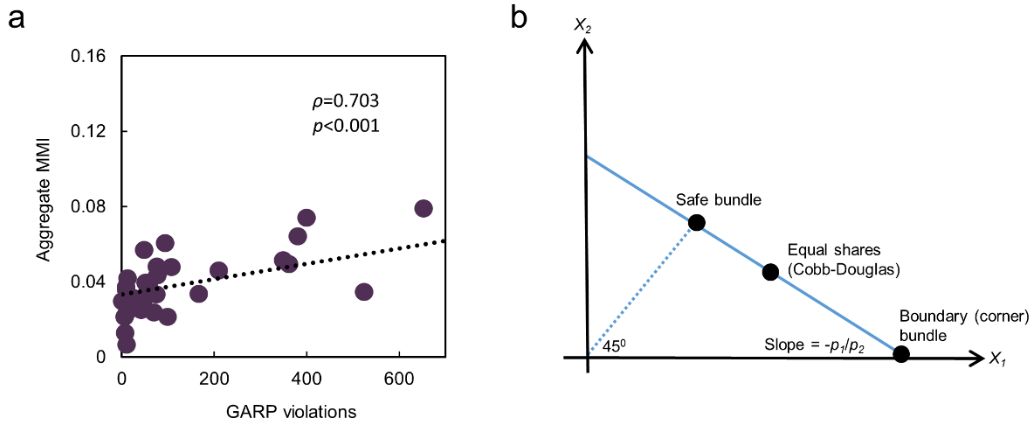

Supplementary Figure 3 | Additional behavioral results. (a) Correlation of the number of GARP violations with Aggregate MMI (ρ=0.703, p<0.001, one data point exceeds the axes). (b) Focal behaviors in the task. Subject may choose to allocate all tokens to the cheaper corner, or, on the other hand, may always choose the safe bundle on the 45 degrees line. Cobb Douglas preferences (with equal exponents) translate into equal share (expenditure) of income on both products, meaning allocating tokens between products proportionally to the price ratio.

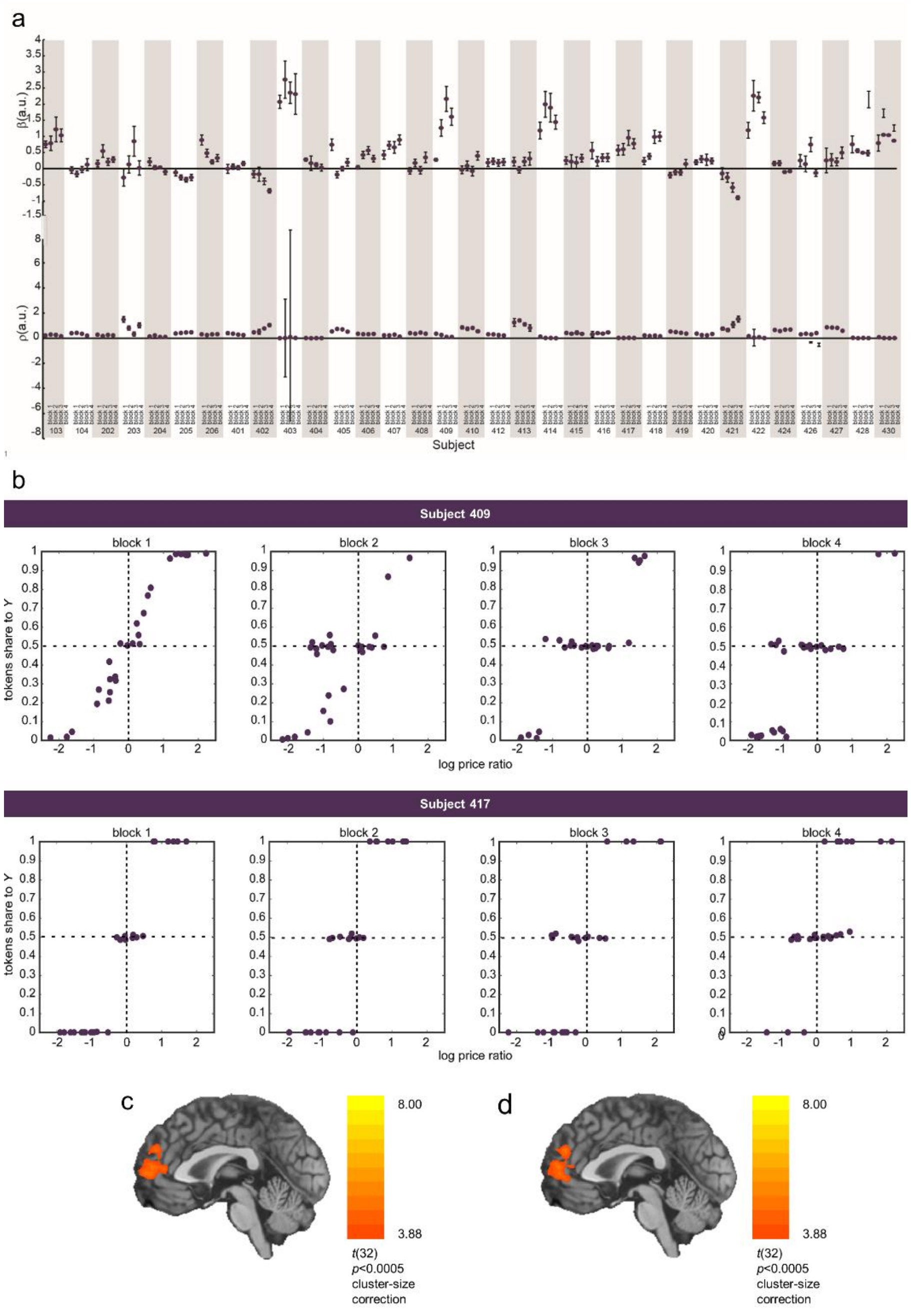

Supplementary Figure 4 | Trial-specific-MMI-blocks. (a) The elicited preferences parameters of DA function with CRRA for each subject in each block with their CI (see footnote 34 in Halevy et al. (2018)[7]. Upper panel shows the recovered β parameter, and bottom panel shows the recovered ρ parameter. The data used to create this figure has been uploaded online (https://osf.io/8jdfh/). (b) Scatterplots of subjects' choice behavior across blocks for two subjects: 417 and 409. The *Y*-axis represents the share of tokens to the *Y*-product, as a function of the log price ratio (slopes). Subject 417 did not change behavior across blocks, while Subject 409 changed behavior: In the first 2 Blocks, she demonstrated Cobb Douglas preferences, and in the 3rd and 4th Blocks she chose similar to Subject 417. (c) We used Trial-specific-MMI-blocks and SV-blocks as predictors for all our subjects. RFX GLM, *n*=33, *p*<0.0005, cluster size correction *x*=0 (MNI coordinates). Model regression: $\mathrm{BOLD} = \beta_0 + \beta_1 \mathrm{RT} + \beta_2 \mathrm{MMI}_{\mathrm{trial\_specific\_blocks}} + \beta_3 \mathrm{SV}_{\mathrm{blocks}} + \beta_4 \mathrm{price\ ratio} + \beta_5 \mathrm{endowment} + \varepsilon$. (d) We repeated the same RFX GLM, this time using Trial-specific-MMI-blocks *and* SV-blocks just with the eight subjects whose behavior changed dramatically over the course of the entire experiment. We used Trial-specific-MMI and SV for the rest of the twenty-five subjects, *p*<0.0005, cluster size correction.

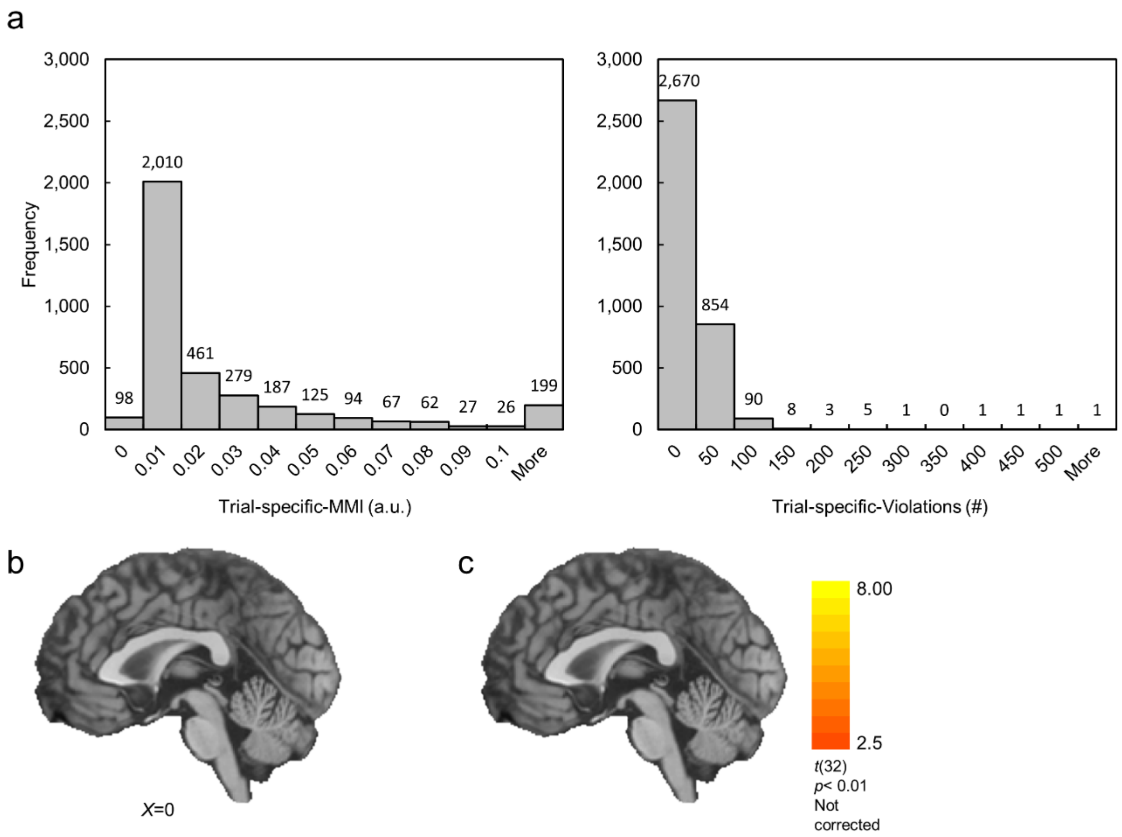

Supplementary Figure 5 | Motivation for using Trial-specific-MMI. (a) Distributions of Trial-specific-MMI vs. Trial-specific-Violations. The latter suffers from low variability in regressor values, as 73.4% of observations equal 0. (b) Nonparametric regressor Trial-specific-Violations. RFX GLM, *n*=33, *p*<0.01, not corrected, *x*=0 (MNI coordinates). Model regression: $\mathrm{BOLD} = \beta_0 + \beta_1 \mathrm{RT} + \beta_2 \mathrm{Violations}_{\mathrm{trial\_specific}} + \beta_4 \mathrm{price\ ratio} + \beta_5 \mathrm{endowment} + +\varepsilon$. (c) Nonparametric regressor Trial-specific-Afriat. RFX GLM, *n*=33, *p*<0.01, not corrected, *x*=0 (MNI coordinates). Model regression: $\mathrm{BOLD} = \beta_0 + \beta_1 \mathrm{RT} + \beta_2 \mathrm{Afriat}_{\mathrm{\_trial\_specific}} + \beta_4 \mathrm{price\ ratio} + \beta_5 \mathrm{endowment} + \varepsilon$.

a

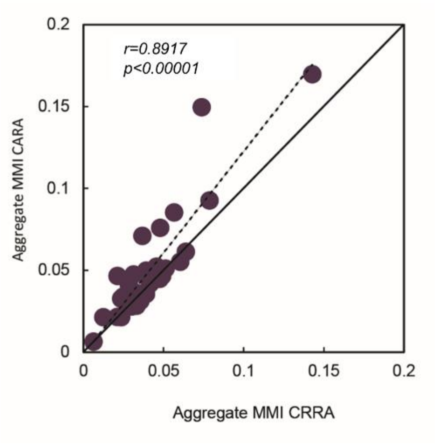

b

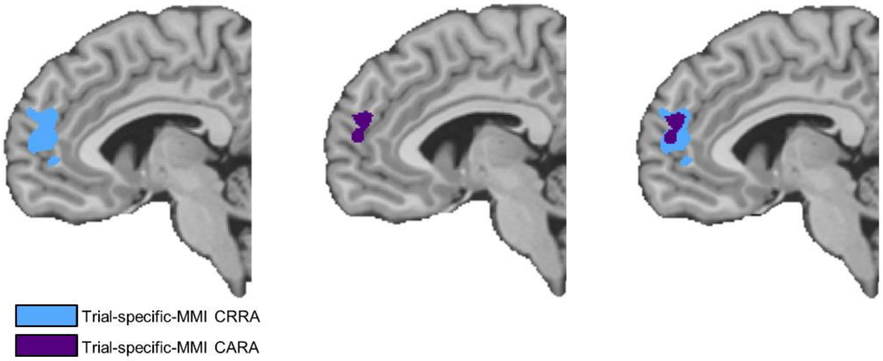

Supplementary Figure 6 | Controlling for misspecification of utility. (a) Correlation of Aggregate MMI using CRRA utility function with Aggregate MMI using CARA utility function (r=0.896, p<0.00001). Most subjects fall above the 45 degrees line, which means using CARA utility function yields a bigger misspecification element compared to the MMI with CRRA function, resulting in higher MMI scores. This result is compatible with the results reported in Halevy et al. (2018)[7]. (b) Choice inconsistency is correlated with vmPFC/ACC activation, regardless the assumption we make about functional form. Results of RFX GLM using CARA functional form, n=33, p<0.0005, cluster-size corrected, x=-5 (MNI coordinates). Model regression: $\text{BOLD} = \beta_0 + \beta_1 \text{RT} + \beta_2 \text{MMI}_{\text{trial-specific-CARA}} + \beta_3 \text{SV}_{\text{CARA}} + \beta_4 \text{price ratio} + \beta_5 \text{endowment} + \varepsilon$.

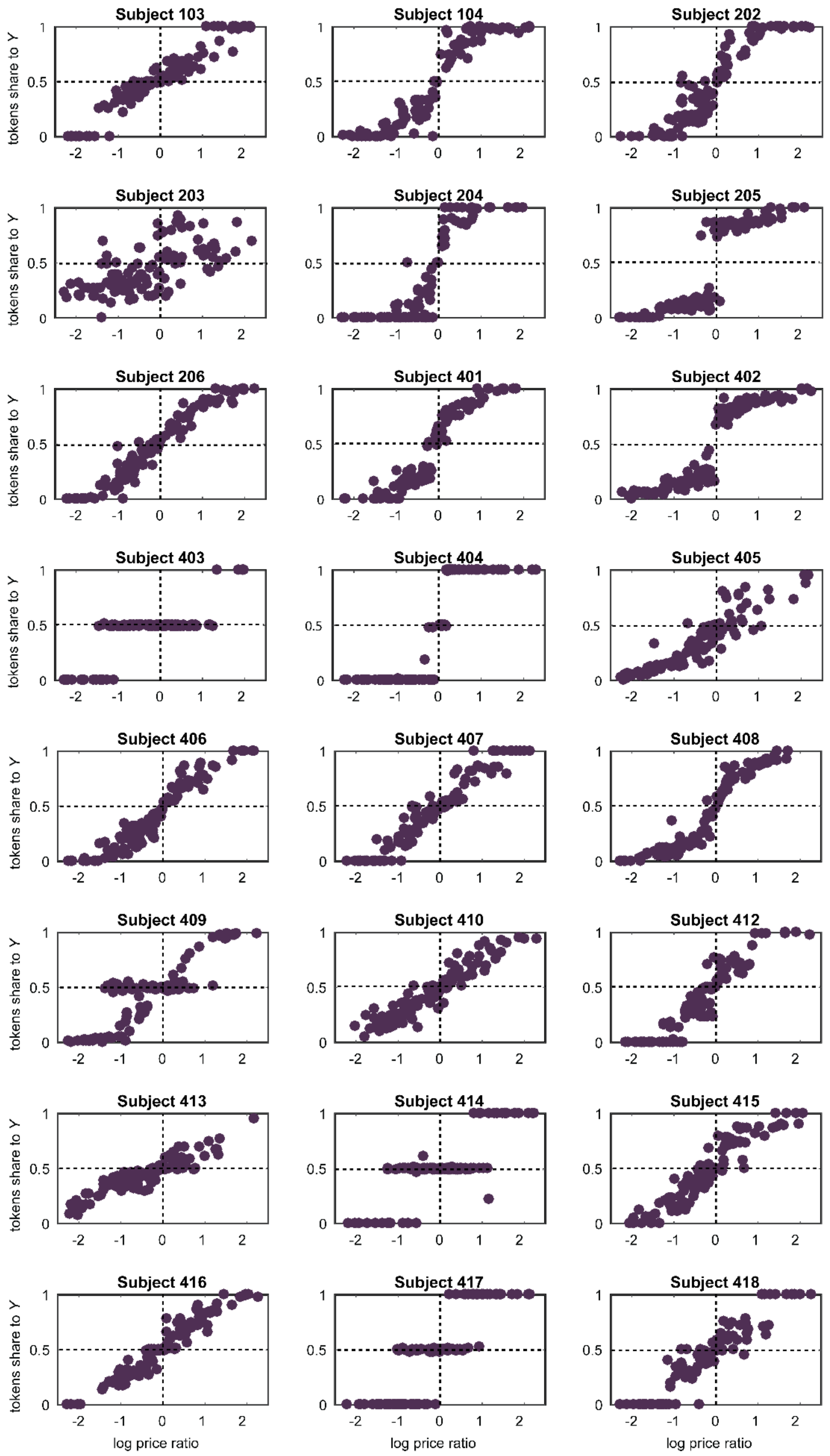
Subject 103
Subject 104
Subject 202
Subject 203
Subject 204
Subject 205
Subject 206
Subject 401
Subject 402
Subject 403
Subject 404
Subject 405
Subject 406
Subject 407
Subject 408
Subject 409
Subject 410
Subject 412
Subject 413
Subject 414
Subject 415
Subject 416
Subject 417
Subject 418
tokens share to Y
log price ratio

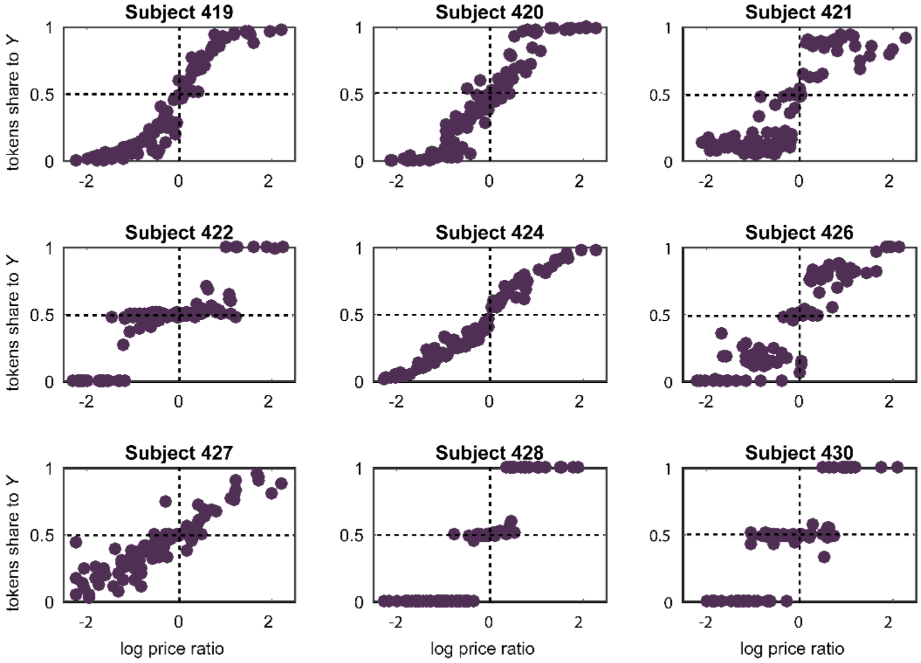

Supplementary Figure 7 | Individual scatterplots of subjects' choices. The *Y*-axis represents the share of tokens allocated to the *Y* product, while the *X*-axis represents the log of price ratio (the slope of the budget set).

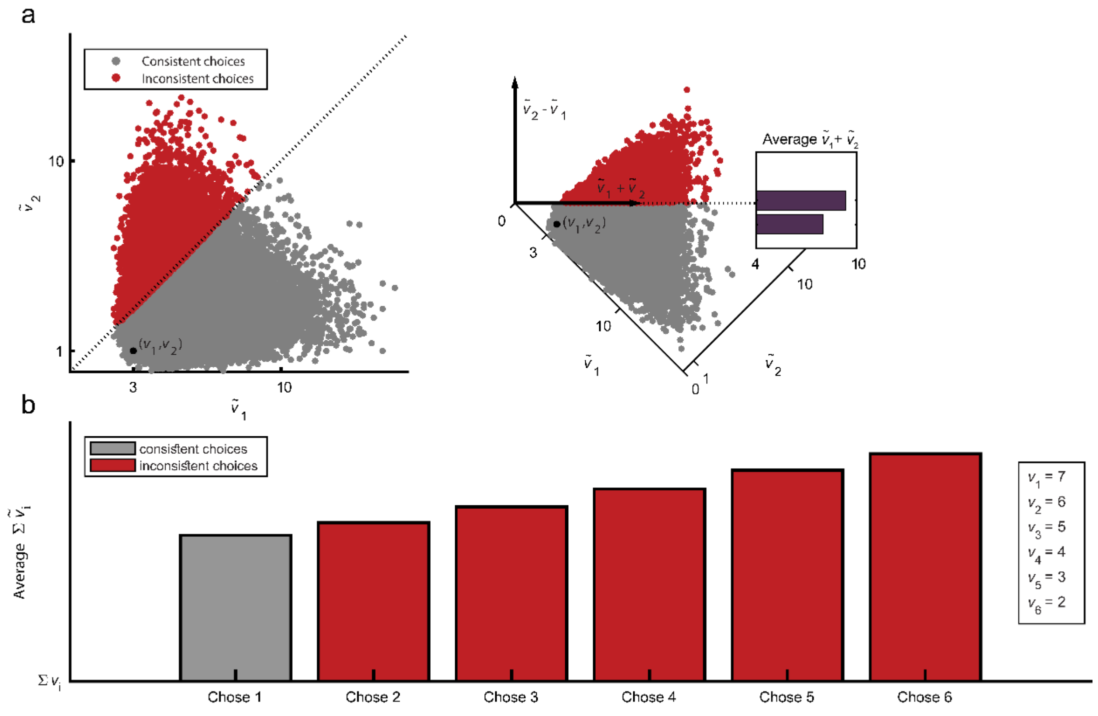

Supplementary Figure 8 | Aggregate Utility in an NRUM is higher for inconsistent choices (a) A sample of 2,000,000 random utilities from Generalized Extreme Value distributions (Type II, Fréchet distribution) with means $v_1 = 4$ and $v_2 = 2$, scale=0.5, location=2. The average aggregate utility is higher when the inconsistent option is chosen. (b) The average aggregate utility increases when the number of alternatives increases (e.g. *N*=6) and is larger when the choice is more inconsistent (e.g. Chose 6 vs. Chose 2).

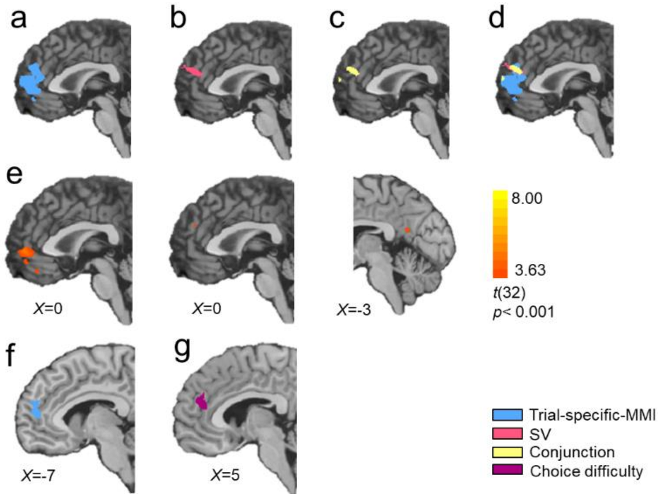

Supplementary Figure 9 | Controlling for task difficulty. (a-d) Replication of Fig. 5, this time the model also controls for the task difficulty. RFX GLM, *n*=33, *p*<0.001, cluster size correction, *x*=0 (MNI coordinates). Model regression: $\mathrm{BOLD} = \beta_0 + \beta_1 \mathrm{RT} + \beta_2 \mathrm{MMI}_{\mathrm{trial\_specific}} + \beta_3 \mathrm{SV} + \beta_4 \mathrm{priceratio} + \beta_5 \mathrm{endowment} + \ \beta_6 \mathrm{choice\_simplicity}$. 6 additional motion-correction regressors were included as regressors of no interest. (a) Neural correlates of the MMI. (b) Neural correlates of SV. (c) Conjunction analysis. (d) Overlay. (e) Neural correlates of Trial-specific-MMI with vmPFC, dACC and PCC activations in a ROI analysis, controlling for choice difficulty. RFX GLM, *n*=33, *q*(FDR)<0.05, MNI coordinates. For illustration purposes, we set the threshold as the whole-brain analysis. Model regression: $\mathrm{BOLD} = \beta_0 + \beta_1 \mathrm{RT} + \beta_2 \mathrm{MMI}_{\mathrm{trial\_specific}} + \beta_3 \mathrm{SV} + \beta_4 \mathrm{priceratio} + \beta_5 \mathrm{endowment} + \beta_6 \mathrm{choice\_simplicity}$. 6 additional motion-correction regressors were included as regressors of no interest. (f-g) Repeating the analysis, excluding the SV and endowment predictors, due to high collinearity with the Choice Simplicity index (R^2=0.5225, clustered regression). *n*=33, *p*<0.0005, cluster size correction. Model regression: $\mathrm{BOLD} = \beta_0 + \beta_1 \mathrm{RT} + \beta_2 \mathrm{MMI}_{\mathrm{trial\_specific}} + \beta_3 \mathrm{priceratio} + \beta_4 \mathrm{choice\_simplicity}$. (f) Neural correlates of the MMI. (g) Our Choice Simplicity index is correlated with ACC activation.

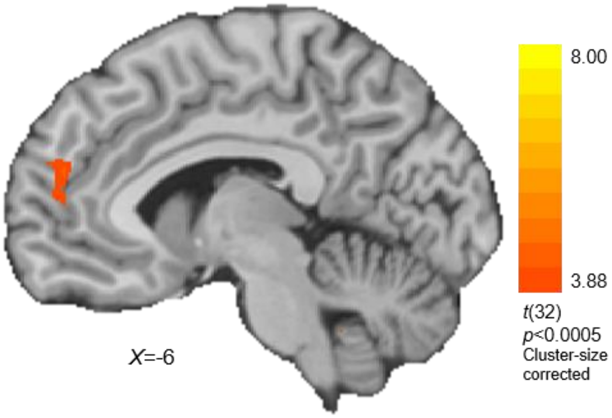

Supplementary Figure 10 | Controlling for the role of confidence in decision-making. Confidence levels were modeled as the second-polynomial order of value (SV-square). The neural correlates of choice inconsistency are not affected by controlling for confidence. RFX GLM, *n*=33, *p*<0.0005, cluster size correction. Model regression: $\mathrm{BOLD} = \beta_0 + \beta_1\mathrm{RT} + \beta_2\mathrm{MMI}_{\mathrm{trial\_specific}} + \beta_3\mathrm{SV} + \beta_4\mathrm{priceratio} + \beta_5\mathrm{endowment} + \ \beta_6\mathrm{SV\_sqr}$.

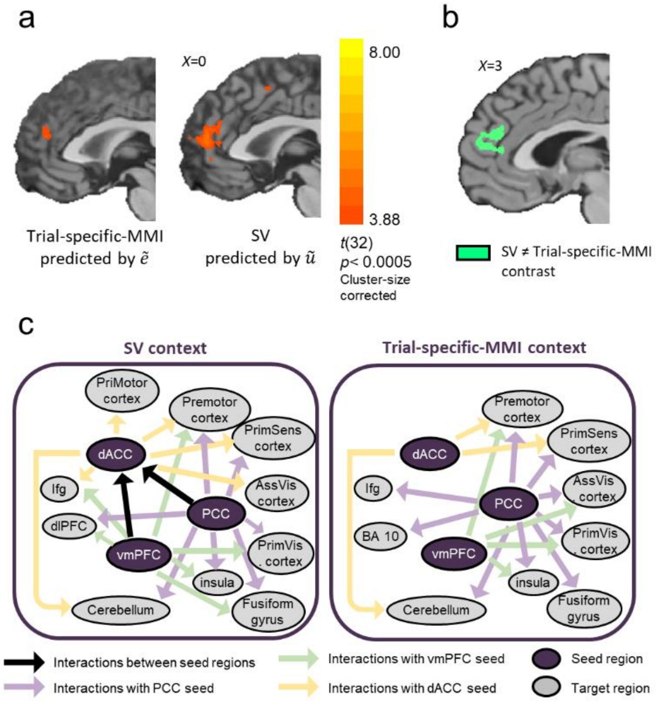

Supplementary Figure 11 | Dissociation of Trial-specific-MMI and SV. (a) Orthogonality analysis. Residuals $\tilde{e}$ used to predict Trial-specific-MMI (*r* = 0.9749, *p*<0.0001), and used as a substitute regressor in an RFX-GLM [*n*=33, RFX, *p*<0.0005, cluster-size correction, model: $\text{BOLD} = \beta_0 + \beta_1\text{RT} + \beta_2\tilde{e} + \beta_3\text{SV} + \beta_4\text{priceratio} + \beta_5\text{endowment}$, 6 additional motion-correction regressors were included as regressors of no interest]. Residuals $\tilde{u}$ used to predict SV (*r* = 0.9749, *p*<0.0001) in RFX-GLM [*n*=33, RFX, *p*<0.0005, cluster-size correction, model: $\text{BOLD} = \beta_0 + \beta_1\text{RT} + \beta_2\text{MMI}_{\text{Trial\_specific}} + \beta_3\tilde{u} + \beta_4\text{priceratio} + \beta_5\text{endowment}$, 6 additional motion-correction regressors were included as regressors of no interest]. (b) Contrast analysis. We defined the two-sided contrast SV≠Trial-specific-MMI, using the same RFX-GLM reported in the whole-brain analysis (Fig. 4(a-d), *p*<0.0005, cluster-size correction). (c) PPI analysis. Results of RFX-GLM, *n*=33, *p*<0.0005, cluster-size correction, MNI coordinates. For each seed region (vmPFC, dACC and PCC) we ran the following models: (1) $\text{BOLD} = \beta_0 + \beta_1\text{Signal}_{seed} + \beta_2\text{SV} + \beta_3\text{PPI} + \beta_4\text{RT} + \beta_5\text{priceratio} + \beta_6\text{endowment}$; (2) $\text{BOLD} = \beta_0 + \beta_1\text{Signal}_{seed} + \beta_2\text{MMI}_{\text{trial\_specific}} + \beta_3\text{PPI} + \beta_4\text{RT} + \beta_5\text{priceratio} + \beta_6\text{endowment}$,6 additional motion-correction regressors were included as regressors of no interest.

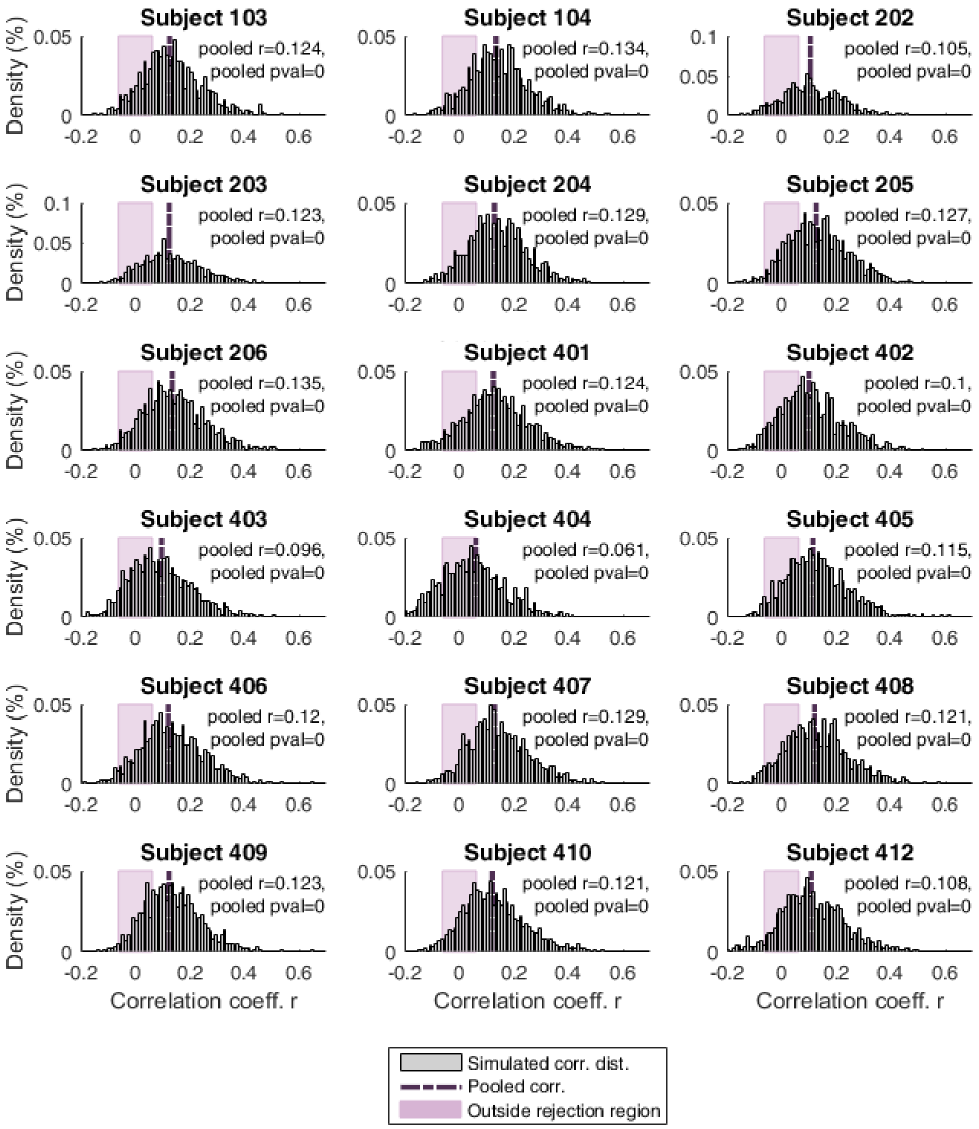
Subject 103
pooled r=0.124, pooled pval=0
Subject 104
pooled r=0.134, pooled pval=0
Subject 202
pooled r=0.105, pooled pval=0
Subject 203
pooled r=0.123, pooled pval=0
Subject 204
pooled r=0.129, pooled pval=0
Subject 205
pooled r=0.127, pooled pval=0
Subject 206
pooled r=0.135, pooled pval=0
Subject 401
pooled r=0.124, pooled pval=0
Subject 402
pooled r=0.1, pooled pval=0
Subject 403
pooled r=0.096, pooled pval=0
Subject 404
pooled r=0.061, pooled pval=0
Subject 405
pooled r=0.115, pooled pval=0
Subject 406
pooled r=0.12, pooled pval=0
Subject 407
pooled r=0.129, pooled pval=0
Subject 408
pooled r=0.121, pooled pval=0
Subject 409
pooled r=0.123, pooled pval=0
Subject 410
pooled r=0.121, pooled pval=0
Subject 412
pooled r=0.108, pooled pval=0
Density (%)
Correlation coeff. r
Simulated corr. dist.
Pooled corr.
Outside rejection region

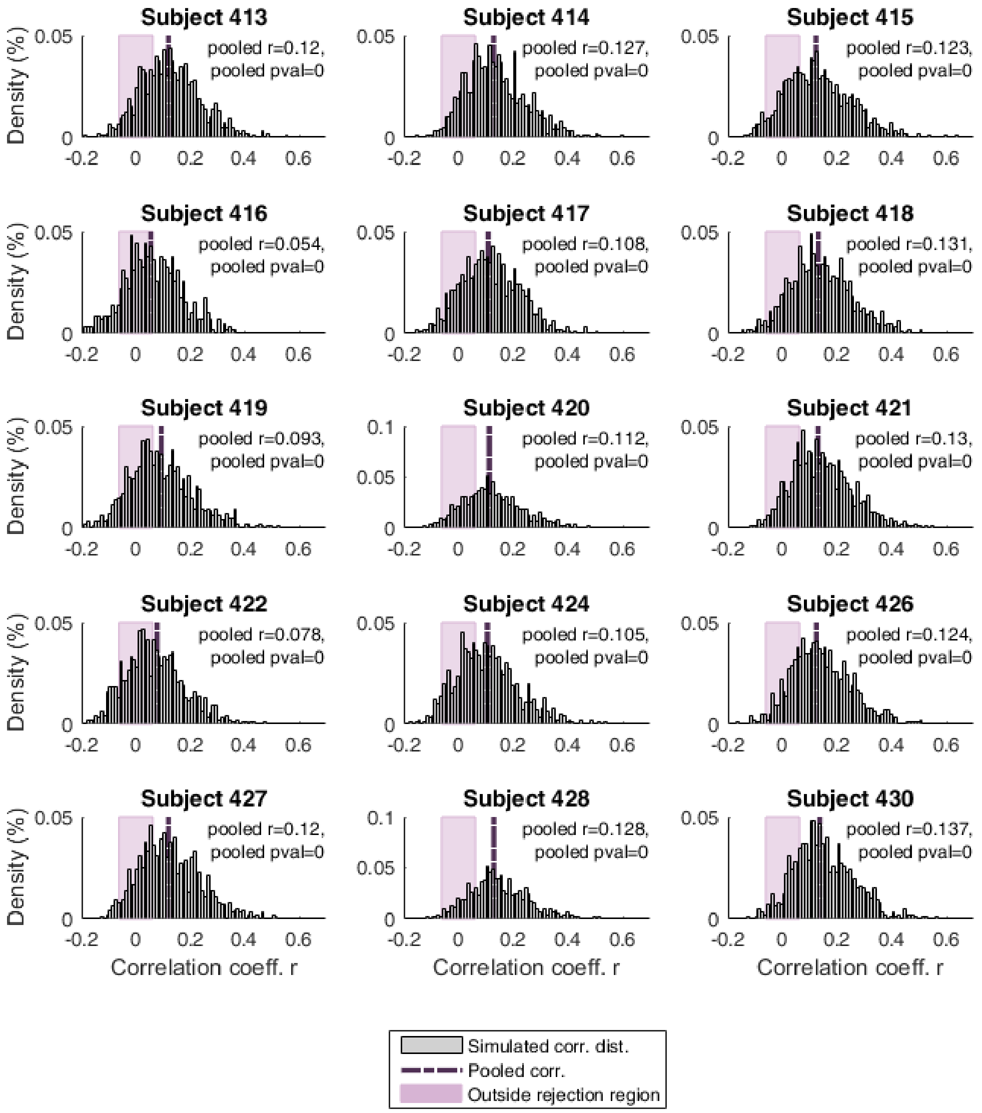

Supplementary Figure 12 | Connecting the NRUM model with observed data. Subject-specific distributions of the correlation between the simulated noise element $\tilde{e}$ of the chosen bundle by the NRUM (with actual elicited parameters on actual decision problems) and the Trial-specific-Afriat, using zero-mode GEV distribution (scale calibrated by actual Afriat index). We get very similar subject specific distributions for the zero-mean log-normal distribution.

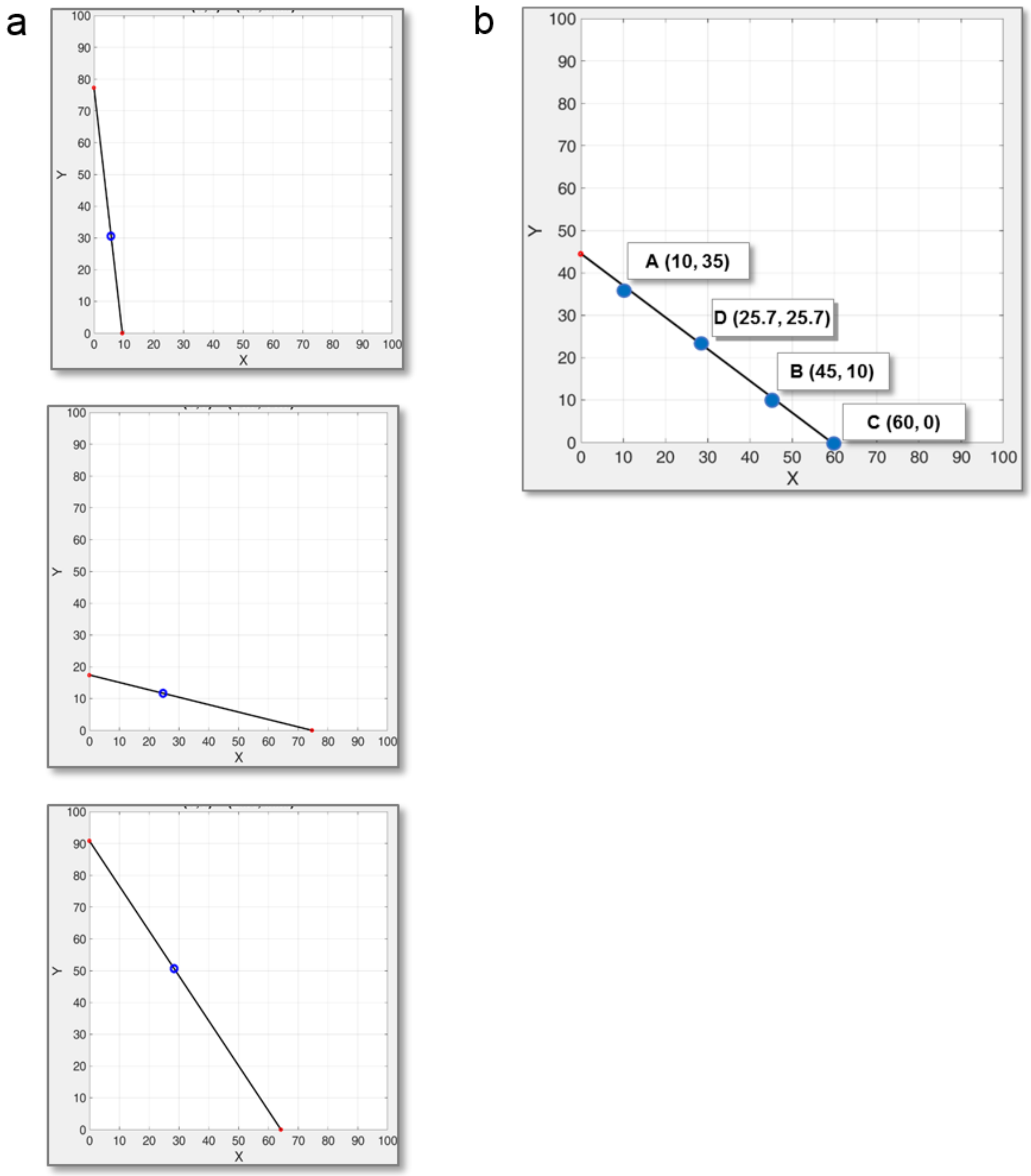

Supplementary Figure 13 | Experimental instructions. (a) Examples for different decision problems. (b) Possible allocations along the line.

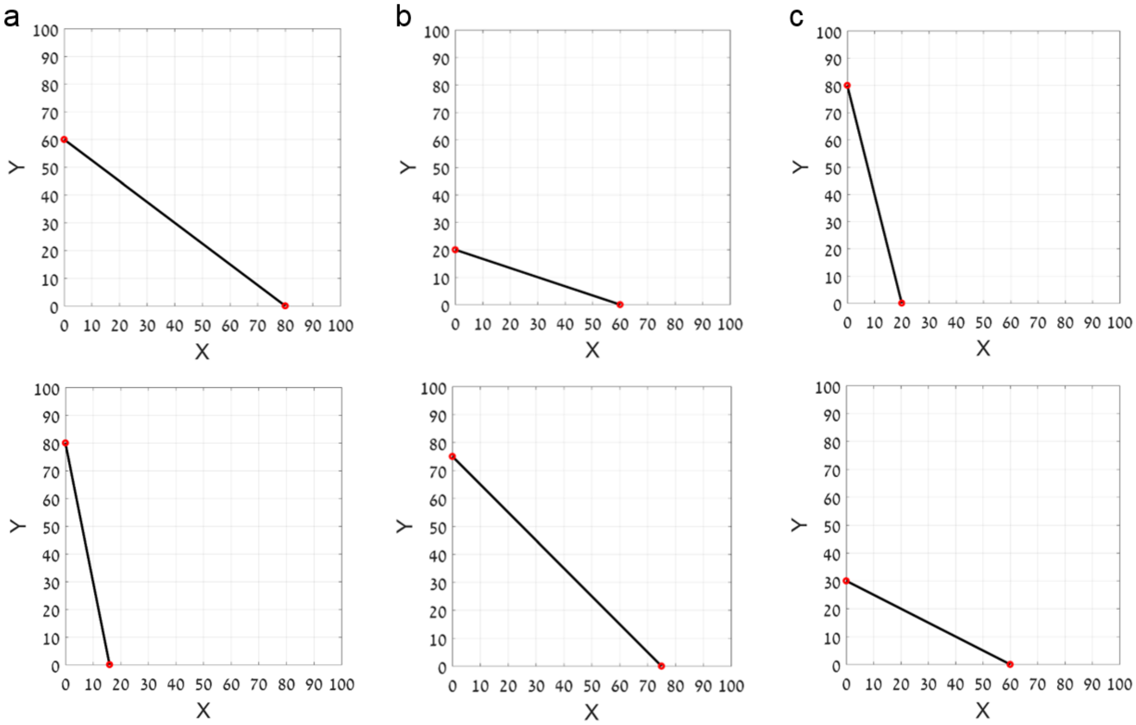

Supplementary Figure 14 | Pre-scan questionnaire. (a) Question 1. Identify intersections with the axes. (b) Question 2. Identify the product that provides you with more tokens. (c) Question 3. Choose a possible allocation of X and Y, and explain what are the possible payoffs for this allocation.

Supplementary Tables

SID	Block 1	Block 2	Block 3	Block 4	Changed behavior?
103	sigmoid	sigmoid	sigmoid	sigmoid	no
104	sigmoid	sigmoid	sigmoid	sigmoid	no
202	sigmoid	sigmoid	sigmoid	sigmoid	no
203	N/D	N/D	N/D	N/D	yes
204	sigmoid	sigmoid	sigmoid	corners	yes
205	corners	corners	corners	corners	no
206	Cobb Douglas	Cobb Douglas	Cobb Douglas	Cobb Douglas	no
401	sigmoid	sigmoid	sigmoid	sigmoid	no
402	corners	corners	corners	corners	no
403	cutoff	cutoff	cutoff	cutoff	no
404	cutoff	cutoff	cutoff	cutoff	no
405	N/D	N/D	Cobb Douglas	Cobb Douglas	yes
406	sigmoid	sigmoid	sigmoid	sigmoid	no
407	sigmoid	sigmoid	sigmoid	sigmoid	no
408	sigmoid	sigmoid	sigmoid	sigmoid	no
409	sigmoid	sigmoid+equal shares	cutoff	cutoff	yes
410	Cobb Douglas	Cobb Douglas	Cobb Douglas	Cobb Douglas	no
412	sigmoid	sigmoid	sigmoid	sigmoid	no
413	equal shares	Cobb Douglas	Cobb Douglas	Cobb douglas	yes
414	cutoff	cutoff	cutoff	cutoff	no
415	sigmoid	sigmoid	sigmoid	sigmoid	no
416	sigmoid	sigmoid	sigmoid	sigmoid	no
417	cutoff	cutoff	cutoff	cutoff	no
418	sigmoid	sigmoid	sigmoid	sigmoid	no
419	sigmoid	sigmoid	sigmoid	sigmoid	no
420	sigmoid	sigmoid	sigmoid	sigmoid	no
421	Cobb Douglas	Cobb Douglas	cutoff	cutoff	yes
422	sigmoid	cutoff	cutoff	cutoff	yes
424	Cobb Douglas	Cobb Douglas	Cobb Douglas	Cobb Douglas	no
426	N/D	N/D	N/D	N/D	yes
427	Cobb Douglas	Cobb Douglas	Cobb Douglas	Cobb Douglas	no
428	cutoff	cutoff	cutoff	cutoff	no
430	cutoff	cutoff	cutoff	cutoff	no

Supplementary Table 1 | Subjects behavior across blocks. When the behavior is not definitive, we classify it as N/D. 8 of our 33 subjects changed behavior across the four blocks of the experiments. By “sigmoid” preferences we refer to Cobb-Douglass preferences where subjects chose corner allocations in steeper slopes. By “cutoff” preferences we refer to choosing the safe bundle for low price ratio and corners for high price ratios.

SID	Avergae Aggregate-MMI-Blocks	Aggregate-MMI entire experiment	difference
203	0.1195	0.1430	0.0235
405	0.0307	0.0481	0.0174
409	0.0346	0.0514	0.0168
418	0.0317	0.0478	0.0161
421	0.0601	0.0740	0.0139
204	0.0217	0.0346	0.0129
206	0.0209	0.0333	0.0124
414	0.0520	0.0641	0.0121
426	0.0670	0.0789	0.0118
417	0.0496	0.0606	0.0109
422	0.0199	0.0305	0.0106
402	0.0278	0.0370	0.0092
403	0.0148	0.0238	0.0090
427	0.0479	0.0569	0.0089
428	0.0134	0.0214	0.0080
430	0.0415	0.0494	0.0079
103	0.0343	0.0419	0.0076
407	0.0330	0.0393	0.0063
406	0.0275	0.0334	0.0059
104	0.0400	0.0458	0.0058
401	0.0251	0.0302	0.0052
413	0.0200	0.0249	0.0049
404	0.0018	0.0065	0.0047
424	0.0084	0.0126	0.0043
408	0.0243	0.0284	0.0041
416	0.0261	0.0297	0.0036
412	0.0324	0.0357	0.0034
420	0.0204	0.0237	0.0033
202	0.0362	0.0395	0.0033
419	0.0185	0.0214	0.0029
415	0.0404	0.0432	0.0028
205	0.0367	0.0394	0.0027
410	0.0291	0.0315	0.0024

Supplementary Table 2 | Comparison of Aggregate MMI and Aggregate MMI-blocks. The average of Aggregate MMI-blocks, which was calculated based on the 27 trials in each block, compared with the Aggregated MMI, which was calculated based on the 108 trials of the experiment. Subjects are sorted in a descending order according to the difference between Aggregate MMI of the entire experiment, and the average of the block indices. Most of the subjects (6 out of 8) who changed behaviors are ranked at the top of the table.

	# of GARP violations	Afriat Index	Aggregate MMI
Left vStr	-0.200	-0.1805	-0.2411
	(0.2645)	(0.3148)	(0.1765)
Right vStr	-0.2733	-0.1367	-0.2071
	(0.1238)	(0.4481)	(0.2476)
vmPFC	-0.31	-0.41	-0.1093
	(0.0791)	(0.0178)	(0.5448)
dACC	-0.1127	-0.2363	-0.0661
	(0.5324)	(0.1855)	(0.7147)
PCC	-0.369	-0.3469	-0.3391
	(0.0346)	(0.0479)	(0.0535)
MMI ROI	-0.1989	-0.2971	0.0159
	(0.267)	(0.0931)	(0.9301)

Supplementary Table 3 | Between-subjects' analysis (*n*=33). We correlated all aggregate indices (Afriat Index, number of GARP violations and Aggregate MMI) with the average change in BOLD signal in our predefined ROIs: vmPFC, vStr (left and right), dACC and PCC. In addition, we correlated the aggregate indices with the average change in BOLD signal in the mPFC/ACC cluster that was correlated with the Trial-specific-MMI ("MMI ROI"). We did not find significant correlations, after correcting for multiple comparisons (Benferroni corrected for 18 independent comparisons, $p_i \leq \frac{0.05}{18} = 0.0028$). The table presents Spearman's ρ (*p*-values).

Condition	Predictor	Region	*x*	*y*	*z*	*t*
Whole-brain (CRRA)	Trial-specific-MMI	mPFC/ACC	0	47	25	5.21
	SV	mPFC/ACC	7	56	16	5.57
		l premotor cortex	6	11	46	5.09
		l associative vis. cortex	-12	-79	37	5.38
		l ventral occipital cortex	-18	-49	-17	5.95
		r pimary motor cortex	39	-22	61	5.08
ROI vmPFC*	Trial-specific-MMI		0	50	1	4.63
	SV		0	47	-2	4.91
ROI dACC*	Trial-specific-MMI		-3	47	24	4.76
	SV		0	42	22	4.76
ROI PCC**	Trial-specific-MMI		-3	-55	19	4.21
	SV		-6	-55	19	4.08
Whole-brain	Trial-specific-MMI	mPFC/ACC	-6	53	10	5.25
w. choice difficulty***	SV	mPFC/ACC	3	44	19	5.25
Whole-brain	Trial-specific-MMI	mPFC/ACC	-6	50	10	5.12
w. choice difficulty,	Choice Difficulty	ACC	0	41	16	5.18
w.o. SV and endowment		r BA 40	63	-22	36	7.15
		l BA 40	-54	-27	37	5.76
		PCC	-3	-28	28	5.91
		l premotor cortex	-3	2	52	5.31
		V1	0	-67	10	5.52
		r fusiform gyrus	49	-64	1	6.16
		l fusiform gyrus	-30	-40	-14	5.7
Whole-brain<br>w. confidence	Trial-specific-MMI	mPFC/ACC	-6	50	19	4.52
Whole-brain (CARA)	Trial-specific-MMI-CARA	mPFC/ACC	-7	50	19	4.390
	SV-CARA	mPFC/ACC	3	44	19	5.25
		Striatum	9	-1	10	5.61
		SMA	3	11	43	5.73
		ventral occipital cortex	-21	-52	-17	7.82
		r premotor cortex	42	-16	55	5.26
		r associative vis. cortex	45	-70	17	5.24
		l associative vis. cortex	-38	-70	4	5.22
Whole-brain	Trial-specific-MMI-blocks	mPFC/ACC	-3	50	10	5.63
(blocks, all subjects)		l dlPFC	-52	38	19	4.58
	SV-blocks	mPFC/ACC	-3	53	13	5.14
		PCC	-3	-52	13	4.92
		r premotor cortex	6	11	43	5.44
Whole-brain	Trial-specific-MMI	mPFC/ACC	-6	50	10	5.29
(blocks, only	SV	mPFC/ACC	-3	50	7	5.39
subjects who		Striatum	9	-1	13	5.2
changed strategies)		PCC	-3	-58	10	5.68
		r premotor cortex	6	11	43	5.73
		r primary motor cortex	42	-22	61	4.89
		r BA7	21	-76	46	4.95
		l associative visual cortex	-9	-79	37	4.98
		l fusiform gyrus	-18	-49	-17	5.4
Motor Localizer****	Motor imprecision	r inferior frontal gyrus	42	17	25	5.02

Supplementary Table 4 | List of neural activations (corrected for multiple comparisons). *x*,*y*,*z* indicate peak voxel coordinates in MNI space, *t* indicates t-statistic in the RFX-GLM. Unless otherwise mentioned, all activations survive multiple comparisons correction of $p<0.0005$, cluster-size corrected with 10 continuous voxels. * *p*(Bonferroni)<0.05, 10 continuous voxels. ** Trial-specific-MMI survives a correction of $p<0.0005$, cluster-size corrected with 10 continuous voxels; SV survives a correction of *q*(FDR)<0.05 with 10 continuous voxels. *** $p<0.001$, cluster-size corrected with 10 continuous voxels. **** $p<0.005$, cluster-size corrected with 10 continuous voxels.

SID	vmPFC	Cluster size	x	y	z	dACC	Cluster size	x	y	z	PCC	Cluster size	x	y	z
103	V	111	8	52	-3	V	135	2	35	21	V	62	-18	-61	40
104	V	5454	10	42	0	V	243	1	38	19	V	492	-12	-48	40
202	-					-					V	38	0	-61	17
203	V	216	-10	46	-1	V	54	0	37	19	V	69	4	-62	28
204	V	54	-3	42	4	V	1593	1	38	26	V	615	-2	-36	33
205	-					-					-				
206	V	5859	10	40	0	V	2997	2	36	23	V	5701	-9	-59	10
401	V	27	10	41	0	V	108	-5	32	22	V	120	-3	-46	13
402	-					-					-				
403	-					V(p<0.2)	27	-9	44	25	V	991	6	-55	10
404	V	3996	10	45	0	V	675	2	34	22	V	3622	12	-42	31
405	V	5508	8	42	2	V	324	3	34	22	V	3565	-12	-37	28
406	-					-					-				
407	V	162	-6	49	-17	V	27	-3	38	16	V	841	15	-46	4
408	V	27	-6	47	4	V	27	0	44	25	V	132	3	-55	19
409	V(p<0.2)	54	-4	47	-8	-					V	17	-12	-55	10
410	-					-					-				
412	-					-					-				
413	V	27	3	38	-8	-					-				
414	V	675	10	55	-7	-					V	88	-7	-61	19
415	V(p<0.2)	27	3	35	-11	-					-				
416	V	27	3	39	-18	V	135	-10	35	33	-				
417	V	378	-1	39	-19	V	27	-3	29	22	V	329	-6	-58	40
418	V	54	-7	41	1	-					V	322	0	-55	6
419	-					-					V	2877	6	-61	10
420	V	810	1	48	3	V	837	2	41	26	V	1799	-3	-52	31
421	V(p<0.2)	54	9	43	1	-					-				
422	-					V(p<0.2)	27	-9	30	29	-				
424	V	918	6	52	-9	V	27	-6	44	31	V	279	3	-37	28
426	V	189	3	46	4	V	625	-9	38	26	V	726	3	-33	34
427	-					-					V	238	-6	-46	22
428	-					-					-				
430	-					V(p<0.2)	27	3	36	22	-				
Total	18 (21)					15 (18)					21				

Supplementary Table 5 | Subject-level conjunction analysis. For each subject, we conducted a conjunction analysis on the brain areas that significantly tracked Trial-specific-MMI and SV. Most subjects had an overlap region in the vmPFC (21 of 33), dACC (18 of 33) and PCC (21 of 33). SID, Subject identification number; (V) indicates there was an overlap region in the vmPFC or dACC; (-) indicates no overlap region in the vmPFC, dACC or PCC. The cluster size is number of anatomical voxels in the ROI. x,y and z are peak voxel coordinates in MNI space. We set a liberal threshold (due to lack of statistical power) of $p<0.15$ for the conjunction probability.

SID	β	ρ
103	0.924	0.241
104	0.020	0.312
202	0.287	0.237
203	0.664	0.491
204	0.096	0.135
205	-0.264	0.435
206	0.476	0.304
401	0.086	0.308
402	-0.211	0.529
403	2.520	0.011
404	0.131	0.025
405	0.111	0.616
406	0.287	0.345
407	0.624	0.232
408	0.105	0.388
409	1.225	0.213
410	0.067	0.750
412	0.209	0.267
413	0.321	0.906
414	1.428	0.059
415	0.269	0.377
416	0.408	0.359
417	0.770	0.024
418	0.667	0.194
419	-0.051	0.432
420	0.257	0.283
421	-0.389	0.813
422	1.888	0.069
424	0.085	0.626
426	0.194	0.357
427	0.370	0.739
428	0.555	0.015
430	0.940	0.030

Supplementary Table 6 | recovered utility parameters. Individual recovered utility parameters for each subject in our sample, using The Disappointment Aversion (DA) utility function with the CRRA utility index[13]**.**

SID	*r*
103	-0.114
104	-0.295***
202	-0.217*
203	-0.369***
204	-0.067
205	-0.229*
206	-0.236*
401	-0.17†
402	-0.185†
403	-0.263**
404	-0.105
405	-0.247**
406	-0.149
407	-0.222*
408	-0.201*
409	-0.258**
410	-0.344***
412	0.075
413	-0.285***
414	-0.378***
415	-0.284***
416	-0.106
417	-0.338***
418	-0.13
419	-0.271***
420	-0.146
421	-0.263**
422	-0.12
424	-0.247**
426	-0.362***
427	-0.408***
428	-0.163†
430	-0.278***

Supplementary Table 7 | Subject-level correlations of Trial-specific-MMI and SV. We find significant negative correlation for 24 out of 33 subjects in our sample. † $p<0.1$, * $p<0.05$, ** $p<0.01$, *** $p<0.005$

#	*X*	*Y*
1	89	8
2	15	71
3	36	36
4	43	5
5	70	4
6	22	35
7	33	33
8	28	26
9	11	42
10	65	7
11	72	14
12	10	62
13	33	15
14	16	27
15	36	44
16	0	70
17	14	41
18	24	24
19	29	32
20	20	37

Supplementary Table 8 | Question 4 in the pre-scan questionnaire. For each winning notification, answer what is the tokens-amount the subject won, and what is the monetary value of the winning prize.

Supplementary References

1. Varian, H. R. The Nonparametric Approach to Demand Analysis. *Econometrica* **50,** 945–973 (1982).
2. Afriat, S. N. The Construction of Utility Functions from Expenditure Data. *Int. Econ. Rev. (Philadelphia).* **8,** 67–77 (1967).
3. Choi, S., Fisman, R., Gale, D. & Kariv, S. Consistency and heterogeneity of individual behavior under uncertainty. *Am. Econ. Rev.* **97,** 1921–1938 (2007).
4. Fisman, R., Kariv, S. & Markovits, D. Individual preferences for giving. *Am. Econ. Rev.* **97,** 1858–1876 (2007).
5. Andreoni, J. & Miller, J. Giving According to GARP: An Experimental Test of The Consistency of Preferences for Altruism. *Econometrica* **70,** 737–753 (2002).
6. Dean, M. & Martin, D. Measuring Rationality with the Minimum Cost of Revealed Preference Violations. *Rev. Econ. Stat.* **98,** 524–534 (2016).
7. Halevy, Y., Persitz, D. & Zrill, L. Parametric Recoverability of Preferences. *J. Polit. Econ.* **126,** 1558–1593 (2018).
8. Varian, H. R. Goodness-of-fit in optimizing models. *J. Econom.* **46,** 125–140 (1990).
9. Karp, R. M. REDUCIBILITY AMONG COMBINATORIAL PROBLEMS. *Complex. Comput. Comput.* 85–103 (1982).
10. Afriat, S. N. On a System of Inequalities in Demand Analysis: An Extension of the Classical Method. *Int. Econ. Rev. (Philadelphia).* **14,** 460–472 (1973).
11. Houtman, M. & Maks, J. A. H. Determining all Maximal Data Subsets Consistent with Revealed Preference. *Kwant. Methoden* **19,** 89–104 (1985).
12. Bronars, S. G. The Power of Nonparametric Tests of Preference Maximization. *Econometrica* **55,** 693–698 (1987).
13. Gul, F. A Theory of Disappointment Aversion. *Econometrica* **59,** 667–686 (1991).
